# Tailored screening and eradication of *Helicobacter pylori* at a subnational level of China: multicenter observational studies in Ya’an city and a rapid meta-analysis in Sichuan province

**DOI:** 10.3389/fpubh.2026.1774904

**Published:** 2026-05-20

**Authors:** Liu Wang, Ou Chen, Xin-Zu Chen, on behalf of the SIGES Research Group

**Affiliations:** 1 Endoscopy Center, Ya’an People’s Hospital - West China Ya’an Hospital, Sichuan University, Ya’an, China; 2 Department of Gastroenterology and Hepatology, Ya’an People’s Hospital - West China Ya’an Hospital, Sichuan University, Ya’an, China; 3 Gastric Cancer Center & Gastric Cancer Laboratory, Department of General Surgery, West China Hospital, Sichuan University, Chengdu, China; 4 Ya’an Cancer Prevention and Control Center, Ya’an People’s Hospital - West China Ya’an Hospital, Sichuan University, Ya’an, China; 5 Ya’an Key Laboratory for High Altitude Medicine, Ya’an People’s Hospital - West China Ya’an Hospital, Sichuan University, Ya’an, China

**Keywords:** antimicrobial resistance, epidemiology, eradication, gastric cancer, *Helicobacter pylori*, prevalence

## Abstract

*Helicobacter pylori* infection remains a major public health concern in China, with prevalence and antimicrobial resistance patterns varying significantly at the subnational level. A multicenter observational study performed in Ya’an city showed that the pooled average prevalence of *Helicobacter pylori* infection was as high as 31.9% (19,058/59,791) during 2021–2023, despite significantly declining form 35.6% in 2021 to 30.4% in 2023 (P for trend < 0.0001). Moreover, men were more likely than woman to be infected in any year (*p* < 0.001) or any age group (*p* < 0.001), with the only exception being the 30–39 years age group. Additionally, the 60–69 years age group had highest prevalence among both males (38.7%) and females (33.5%), respectively. A sequential multicenter study in Ya’an (2023–2024) found high antimicrobial resistance to metronidazole (96.9%), levofloxacin (45.9%), and clarithromycin (30.2%), with 17.6% multi-drug resistance. A rapid meta-analysis on four recent studies in Sichuan province (1,203 patients) found the isolation rate of *Helicobacter pylori* was 70.7% (95% CI 55.8%–85.6%). The pooled estimate consistently confirmed those high resistance rates, although amoxicillin (0.4%), tetracycline (0.3%), and furazolidone (0%) maintained consistently extremely low resistance in Sichuan. Rifampicin resistance was quite diverse in Sichuan. These findings highlight the need for organized massive screening for *Helicobacter pylori* infection that should be encouraged in moderate-to-high prevalence regions at subnational level of China to identify individuals at high risk of gastric cancer. Both antimicrobial resistance prevalence-guided empirical eradication and susceptibility-guided individualized eradication require further investigations on effectiveness and cost-effectiveness to improve eradication of *Helicobacter pylori* infection.

## Introduction

*Helicobacter pylori* (*H. pylori*) infection, which is known as a class-I carcinogenetic pathogen associated with an increased risk of incident gastric cancer, has long been a public concern (1) Active screening and eradication of *H. pylori* are considered effective methods to reduce the incidence of gastric cancer and improve population-level survival (2). Several recent studies reported the prevalence and antimicrobial resistance situation of *H. pylori* in Sichuan province, China (3–5). However, epidemiologic features of *H. pylori* infection are still diverse at the subnational level in China. More robust understanding of the subnational situation could provide more precise recommendations on *H. pylori* screening and eradication. Therefore, we aim to briefly report the relevant results in Ya’an, a city located in the West Sichuan Province.

## Methods

A multicenter observational study including 11 municipal and district/county hospitals was performed between Jan 2021 and Dec 2023 in Ya’an. This study aimed to estimate the prevalence of *H. pylori* infection among the population in Ya’an. The study was approved by the Biomedical Ethical Committee of Ya’an People’s Hospital (identifier: 2024-064) with a waiver of informed consent due to its retrospective nature. The study retrospectively collected observations on eligible participants living in Ya’an, who received ^13^C-urea or ^14^C-urea breath test to detect *H. pylori* infection during their health check-ups. Repeat tests from the same individuals were unavailable. There were no limitations on criteria regarding attendee age, sex, or ethnicity. Overall, sex-specific and age-specific prevalence of *H. pylori* infection were estimated. Meanwhile, their trends were analyzed using the Cochran-Armitage trend test, and P for trend (P*_Trend_*) was estimated.

A sequential multicenter observational study including seven municipal and district/county hospitals was performed between Nov 2023 and Apr 2024 to evaluate the antimicrobial resistance of *H. pylori* in Ya’an. This study was approved by the Biomedical Ethics Committee of Ya’an People’s Hospital (identifier: 2023-012). Informed consent was obtained from all participants. *H. pylori*-infected patients, detected by urea breath test, were recruited. Gastric mucosa specimens were collected during opportunistic gastroscopies and biopsies. *H. pylori* colony isolation and antimicrobial susceptibility tests were sequentially carried out based on fresh specimens. Antimicrobial resistance rates of eight common antibiotics for *H. pylori* eradication were estimated.

Additionally, a rapid meta-analysis was performed to pool the antimicrobial resistance rates of seven common antibiotics for *H. pylori* eradication based on recent evidence. The PubMed database was searched by strings “*Helicobacter pylori* AND antibiotics AND resistance AND Sichuan”. Observational studies that published extractable data from Sichuan province about the antimicrobial resistance of *H. pylori* in 2025, as well as the above study in Ya’an, were eligible. The temporal trends of antimicrobial resistance rates over time were not considered in the present meta-analysis. Meta-analysis pooling of aggregate data using the random-effects inverse-variance model with DerSimonian-Laird estimate was used. Pooled rates with 95% CI confidence intervals (CIs) of antimicrobial resistance were estimated. The STATA/SE 14.0 for Windows software was used for all the above statistical analyses. Two-sided *p* values less than 0.05 were considered significant.

## Results

**Figure 1:**
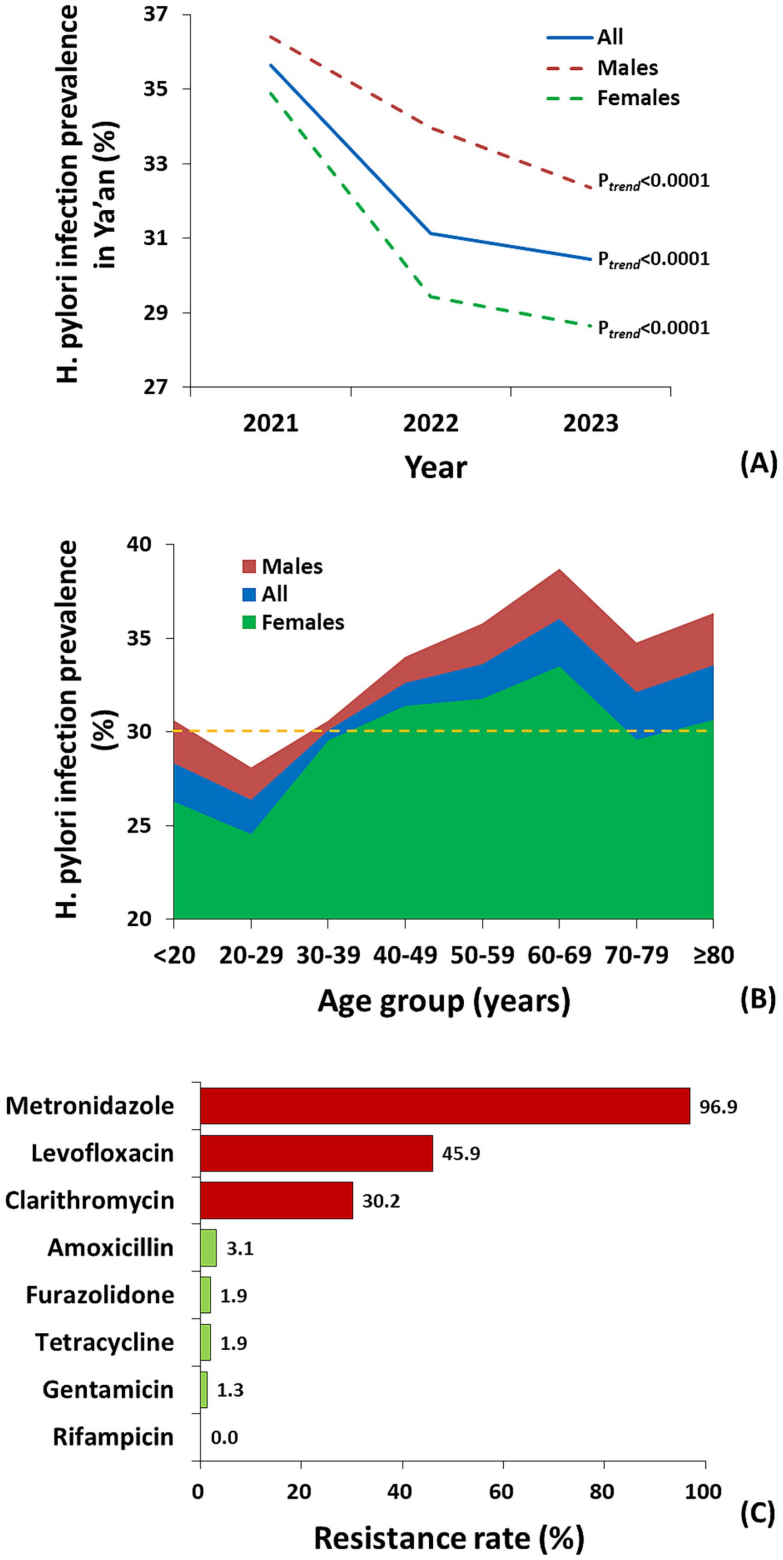
Multicenter studies on *H. pylori* infection prevalence and antimicrobial resistance in Ya’an city. **(A)** Trends of *H. pylori* infection prevalence over time; **(B)** Age-specific *H. pylori* infection prevalence (broken line for cutoff of moderate prevalence, >30%); **(C)** Antimicrobial resistance rates.

In total, 59,791 observations were analyzed and the pooled average prevalence of *H. pylori* infection was 31.9% (19,058/59,791) across the three-year study period (2021–2023). A significantly descending prevalence trend was observed from 2021 (35.6%) to 2023 (30.4%) (P*_trend_* < 0.0001) (Figure 1A). The prevalence among males was always significantly higher than that among females during 2021–2023 or of any age group (*p* < 0.001), with the only exception of age 30–39 years group (Figures 1A,B). Moreover, the decline of prevalence was visually sharper among females over time (Figure 1A). The age-specific prevalence was similar trends in all and sex-specific subpopulation (Figure 1B). Prevalence peaked for the 60–69 years group, at 36.1, 38.7, and 33.5% for whole population, males, and females, respectively (Figure 1B).

Sequentially, 214 *H. pylori*-infected patients were recruited. Finally, 159 colonies of *H. pylori* were successfully obtained out of the biopsies from recruited patients (74.3%). Mono-drug, dual-drug, and multi-drug resistance rates were 37.7, 42.1, and 17.6%, respectively. Metronidazole, levofloxacin, and clarithromycin showed the highest resistance rates of 96.9%, 45.9%, and 30.2%, respectively, significantly exceeding those of other antibiotics (Figure 1C). Antimicrobial resistance rates of amoxicillin, tetracycline, furazolidone, gentamicin, and rifampicin were 3.1, 1.9, 1.9, 1.3, and 0%, respectively (Figure 1C).

**Figure 2:**
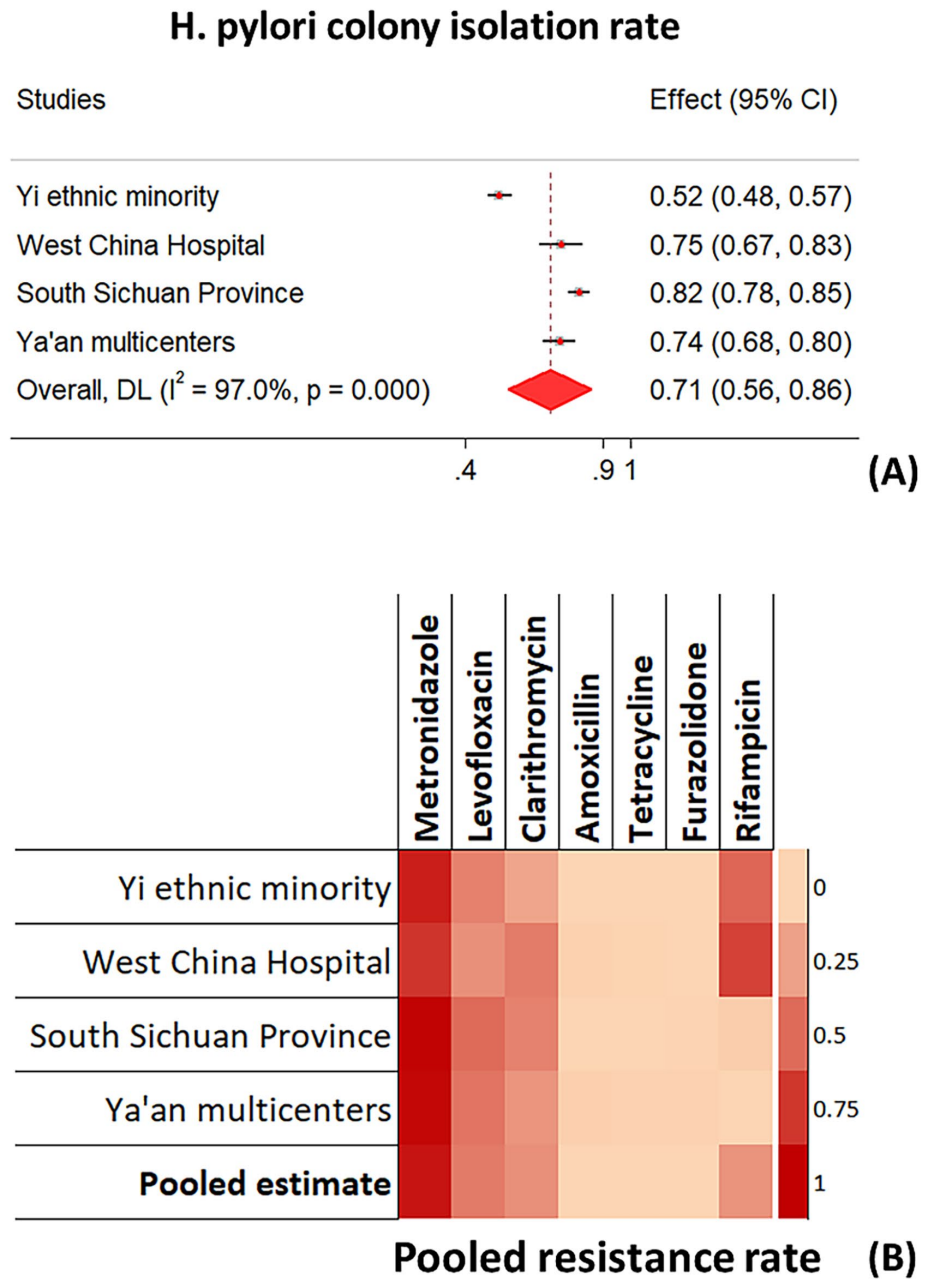
Meta-analyses on **(A)** isolation rate of *H. pylori* colony and **(B)** antimicrobial resistance rate in Sichuan province.

Four eligible observational studies were included in the rapid meta-analysis (3–5), and 1,203 *H. pylori*-infected patients were analyzed. The pooled rate of successfully isolated *H. pylori* colonies was 70.7% (95% CI 55.8–85.6%) (Figure 2A). The pooled antimicrobial resistance rates of metronidazole (90.7, 95% CI 84.1–97.3%), levofloxacin (42.7, 95% CI 35.7–49.6%), and clarithromycin (33.1, 95% CI 24.1–42.1%) ranked top 3, and the rates were consistent among four studies (Figure 2B; Table 1). Conversely, amoxicillin (0.4%), tetracycline (0.3%), and furazolidone (0%) demonstrated negligible resistance rates across all four studies (95% CIs: 0–1.1%, 0–0.8%, 0–0.1%; Figure 2B; Table 1). Additionally, the antimicrobial resistance of rifampicin (30.6, 95% CI 14.4–46.8%) was highly diverse among different areas or subpopulations, while fairly low rates were found in Ya’an and South Sichuan Province (Figure 2B; Table 1).

**Table 1:** Meta-analysis pooling the antimicrobial resistance rates of seven antibiotics in Sichuan province.*

Studies	Metronidazole	Levofloxacin	Clarithromycin	Amoxicillin	Tetracycline	Furazolidone	Rifampicin
Yi ethnic minority (3)	86.3 (82.1–90.5)	39.7 (33.8–45.6)	22.9 (17.8–28.0)	0	0.4 (0–1.2)	0	52.7 (46.7–58.7)
West China Hospital (4)	75.6 (66.5–84.7)	32.6 (22.7–42.5)	41.9 (31.5–52.3)	2.3 (0–5.5)	1.2 (0–3.5)	0	69.8 (60.1–79.5)
South Sichuan Province (5)	98.7 (97.4–100)	50.0 (44.4–55.6)	39.1 (33.7–44.6)	0.3 (0–0.9)	0	1.0 (0–2.1)	4.0 (1.8–6.1)
Ya’an multicenters	96.9 (94.1–99.6)	45.9 (38.2–53.7)	30.2 (23.1–37.3)	3.1 (0.4–5.9)	1.9 (0–4.0)	1.9 (0–4.0)	0
Pooled rate	90.7 (84.1–97.3)	42.7 (35.7–49.6)	33.1 (24.1–42.1)	0.4 (0–1.1)	0.3 (0–0.8)	0 (0–0.1)	30.6 (14.4–46.8)

* Antimicrobial resistance rate: % (95% confidence intervals).

## Discussion

These findings demonstrated that the moderate prevalence of *H. pylori* infection in Ya’an exhibited a similar descending trend to that in Chengdu, the provincial capital city of Sichuan Province, China (6). In the urban health check-up population in Chengdu, the prevalence declined from 53.1% (2009–2010) to 30.7% (2019–2021) (7). Meanwhile, the peak of prevalence shifted to the older age group (60–69 years) in both Chengdu and Ya’an (7). It should be noted that *H. pylori* prevalence in the <20 years age group has increased in recent years. However, the Tibetan and Yi ethnic minorities still had a relatively higher prevalence of *H. pylori* infection, reaching up to 52.3%–62.2% (3, 8–10). Health policies and basic medical insurance should strengthen organized large-scale screening and eradication of *H. pylori* among ethnic minorities in Sichuan Province (11, 12). In the updated guidelines for gastric cancer screening (2024 edition) (13), officially issued for the first time by the National Health Commission of the People’s Republic of China, patients infected with *H. pylori* are defined as a high-risk subpopulation for gastric cancer and are recommended for endoscopic screening and surveillance if they are older than 45 years. Therefore, active eradication of *H. pylori*, combined with organized endoscopic screening and surveillance of high-risk subpopulations, should be systematically considered to control gastric cancer-specific mortality, especially in moderate-to-high prevalence regions (2, 14).

Rising antimicrobial resistance has become a major global challenge in recent decades, driven primarily by inappropriate antibiotic use (15). Between 2010 and 2021, China had the second highest annual growth rate of antibiotic consumption, reaching up to 7% (15). However, high-dose antibiotic treatment remains the mainstream approach for *H. pylori* eradication. The growing challenge of antimicrobial resistance in *H. pylori* underscores the need for susceptibility-guided eradication (16). Current antimicrobial susceptibility tests cannot fully meet clinical requirements, due to the merely mild-to-moderate success rate of *H. pylori* colony isolation (52.3%–81.5%); thus, novel techniques are expected to improve diagnostic performance (16). Although amoxicillin was the most widely used antibiotic (28%) worldwide (15), the antimicrobial resistance rate of *H. pylori* to amoxicillin remains quite low (0%–3.1%) in Sichuan Province. Therefore, amoxicillin is still strongly recommended for empirical treatment in the latest Chinese guidelines (17), and even high-dose amoxicillin-containing dual therapy can be implemented in certain situations (18). Metronidazole, levofloxacin, and clarithromycin consistently exhibit high antimicrobial resistance rates, while rifampicin shows diverse antimicrobial resistance rates in Sichuan Province; rifampicin particularly had no antimicrobial resistance in Ya’an. However, metronidazole, levofloxacin, and clarithromycin are still recommended as first-line agents in bismuth quadruple therapy, whereas rifampicin is not considered in either first-line or second-line therapy (17).

The possible causes of *H. pylori* antimicrobial resistance in Sichuan, consistent with our Ya’an multicenter studies and Sichuan meta-analysis, include unreasonable medication (overuse, misuse, incomplete courses, or arbitrary selection) and strong regional antibiotic selection pressure from widespread clarithromycin and metronidazole use, explaining extremely high metronidazole (96.9%) and clarithromycin (30.2%) resistance in Ya’an. High levofloxacin resistance in Ya’an (45.9%) may result from two mechanisms: spontaneous mutations in target genes (23S rRNA for clarithromycin, gyrA for levofloxacin) under antibiotic pressure (19, 20), and biofilm formation that promotes multidrug resistance (17.6%) (21). Heterogeneous infection and poor patient compliance (missed doses or early discontinuation) further promote resistance, alongside diverse rifampicin resistance across Sichuan. Antimicrobial resistance reduces first-line regimen efficacy and increases treatment failure and reinfection risks, particularly in Sichuan where amoxicillin (0.4%), tetracycline (0.3%), and furazolidone (0%) maintain low resistance. Thus, personalized regimens based on local susceptibility testing (prioritizing low-resistance antibiotics) and complementary measures (rational antibiotic use, improved compliance, and optimized proton pump inhibitor dosing) are recommended. To control resistance, strict rational antibiotic use, adherence to full treatment, selection based on regional/individual susceptibility, reduced unnecessary antibiotic exposure, and strengthened infection control are essential to slow resistant strain emergence, aligned with tailored subnational eradication strategies for gastric cancer control.

Some limitations of the present investigation need to be considered. First, the study population comprised a convenience sample of health check-up attendees, which may not fully represent the general population of Ya’an. For instance, individuals who do not undergo regular health check-ups were not included, which could underestimate the prevalence of *H. pylori*. Second, only demographic features were retrieved and analyzed in this retrospective observation. The potential explanation of declining prevalence in Ya’an was underestimated. Socioeconomic development, public health interventions, and public awareness improvement are important factors influencing the downward prevalence of *H. pylori* in Ya’an, but they cannot be evidenced by the present study design. Third, the urban–rural disparity cannot be evaluated in the present investigation. Urban and rural subpopulations might have different prevalences of *H. pylori* infection, and tailored screening strategies need consider urban–rural disparity. Fourth, the exact rates and details of eradication in separate cross-sections were unavailable and, therefore, no further understanding and evaluation of eradication could be obtained in this study. Last, between-study heterogeneity in meta-analysis might be introduced by diverse subpopulations from different regions. Namely, antimicrobial resistance rate of *H. pylori* is considered with a potential geography-specific bias.

*H. pylori* prevalence in Sichuan Province is moderate overall, but significantly higher among ethnic minorities. Tailored health policies and basic medical insurance support need improvement to strengthen organized massive *H. pylori* screening and eradication in moderate-to-high prevalent areas or subpopulations. In the future, both antimicrobial resistance prevalence-guided empirical eradication and susceptibility-guided individualized eradication require further investigations on effectiveness and cost-effectiveness to improve eradication of *H. pylori* infection and therefore control gastric cancer incidence.

## Data Availability

The raw data supporting the conclusions of this article will be made available by the authors, without undue reservation.
